# Supplementary material for: Postnatal supplementation with alarmins S100a8/a9 ameliorates malnutrition-induced neonate enteropathy in mice
Source: Nat Commun. 2024 Oct 4;15:8623. doi: 10.1038/s41467-024-52829-x (PMC11452687; doi:10.1038/s41467-024-52829-x)
Supplement: Supplementary file 1 — Supplementary Information [file 41467_2024_52829_MOESM1_ESM.pdf]

Supplementary information

Postnatal supplementation with alarmins S100a8/a9 ameliorates malnutrition-induced neonate enteropathy in mice

Lisa Peruzza[1,a,c], Julia Heckmann[2,a], Tanja Rezzonico Jost[1], Matteo Raneri[1], Simone Guglielmetti[3,4], Giorgio Gargari[4], Martina Palatella[1], Maike Willers[5], Beate Fehlhaber[5], Christopher Werlein[6], Thomas Vogl[7], Johannes Roth[7], Fabio Grassi[1,b], and Dorothee Viemann[2,5,8,9,b]

[1] Institute for Research in Biomedicine, Faculty of Biomedical Sciences, Università della Svizzera Italiana (USI), 6500, Bellinzona, Switzerland.

[2] Department of Pediatrics, University Hospital Würzburg, Würzburg, Germany

[3] Department of Biotechnology and Biosciences (BtBs), University of Milano-Bicocca, Milan, Italy

[4] Department of Food, Environmental and Nutritional Sciences (DeFENS), University of Milan, Milan, Italy

[5] Department of Pediatric Pneumology, Allergology and Neonatology, Hannover Medical School, Hannover, Germany

[6] Institute of Pathology, Hannover Medical School, Hannover, Germany

[7] Institute of Immunology, University of Münster, Münster, Germany

[8] Center for Infection Research, University Würzburg, Würzburg, Germany

[9] Cluster of Excellence RESIST (EXC 2155), Hannover Medical School, Hannover, Germany

[a] These authors have contributed equally to this work and share first authorship.

[b] These authors jointly supervised this work and share senior authorship.

[c] Present address: Humabs BioMed SA a Subsidiary of Vir Biotechnology Inc., Bellinzona 6500, Switzerland.

This file includes:

Supplementary Table 1

Supplementary Figure 1-7

Supplementary Table 1 Composition of standard and protein reduced animal chow.

Animal chow	Standard	Protein reduced
Provider	Altromin	ssniff
Order number	1314	S7088-E710
Diet type	grain-based	purified
**Energy**		
Calories (kcal/kg)	3340	3420
Fat (%)	14	6
Protein (%)	27	7
Carbohydrates (%)	59	87
**Proximate contents (%)**		
Crude Ash	6.1	5.9
Crude Fibre	4.5	2.1
Crude Fat	5.1	8.7
Crude protein	22.5	3.4
Nitrogenfree extractives	50.7	-
Starch	35	47
Sugar	5.4	21.4
**Ingredients (g/kg)**		
Casein	-	70
Corn starch	yes	499
Maltodextrin	-	62
Dextrose	-	220
Sucrose	-	2.1
Cellulose	yes	66.3
Inulin	-	22.1
Vitamin premix, AIN93G	yes	8.9
Mineral premix, AIN93G	yes	39.8
Choline bitartrate	-	1.8
Soybean oil	yes	20
Dye	-	0.3
**Carbohydrates (g/kg)**		
Monosaccharides	0	220
Disaccharides	54.2	60
Polysaccharides	350	590
**Minerals and trace elements (mg/kg)**		
Clacium	7062	5000
Potassium	10144	3600
Magnesium	2055	507
Sodium	2154	1020
Phosphorus	5090	3000
Aluminium	81.85	-
Clorine	3382.9	1570
Iron	191.02	35
Flouride	3.05	1
Iodine	1.53	0.2
Cobalt	0.37	-
Copper	13.89	6
Manganese	77.69	10
Molybdenum	1.54	0.15
Sulfur	974.4	1300
Selenium	0.26	0.3
Zinc	84.99	30
Silicium	-	5
Chrome	-	1
Nickel	-	0.5
Boron	-	0.5
Lithium	-	0.1
Vanadium	-	0.1
**Vitamines**		
Vitamin A (IU/kg)	15000	4000
Vitamin D3 (IU/kg)	600	1000
Vitamin E (mg/kg)	77	75
Vitamin K3 (mg/kg)	3	0.75
Vitamin B1 (mg/kg)	18	5
Vitamin B2 (mg/kg)	12	6
Vitamin B6 (mg/kg)	9	6
Vitamin B12 (µg/kg)	24	25
Nicotinic acid (mg/kg)	36	30
Pantothenic acid (mg/kg)	21	15
Folic acid (mg/kg)	2	2
Biotin (µg/kg)	260	200
Choline chloride (mg/kg)	600	-
Vitamin C (mg/kg)	36	-
Alanin (mg/kg)	10284	-
Arginine (mg/kg)	14822	-
Aspartic acid (mg/kg)	21735	-
Cystine (mg/kg)	3244	1000
Glutaminc acid (mg/kg)	43649	-
Cystemin (mg/kg)	9565	-
Hostigin (mg/kg)	5508	-
Isoleucin (mg/kg)	9668	-
Leucin (mg/kg)	17123	-
Lysine (mg/kg)	11326	4800
Methionine (mg/kg)	3171	1800
Phenylalanine (mg/kg)	10549	-
Proline (mg/kg)	13955	-
Serine (mg/kg)	11300	-
Thereonin (mg/kg)	8249	-
Tryptophan (mg/kg)	2929	-
Tyrosine (mg/kg)	7561	-
Valine (mg/kg)	10738	-
**Fatty acids (mg/kg)**		
Arachidic acid (C20:0)	148	-
Eicosanoic acid (C20:1)	185	-
Alpha-Linolenic acid (C18:3)	3018	1200
Linolenic acid (C18:2)	21996	10800
Palmitic acid (C16:0)	5342	2500
Stearic acid (C18:0)	1615	700
Oleic acid (C18:1)	9287	5200

yes, contained, but variable due to the raw materials used or a trade secret.

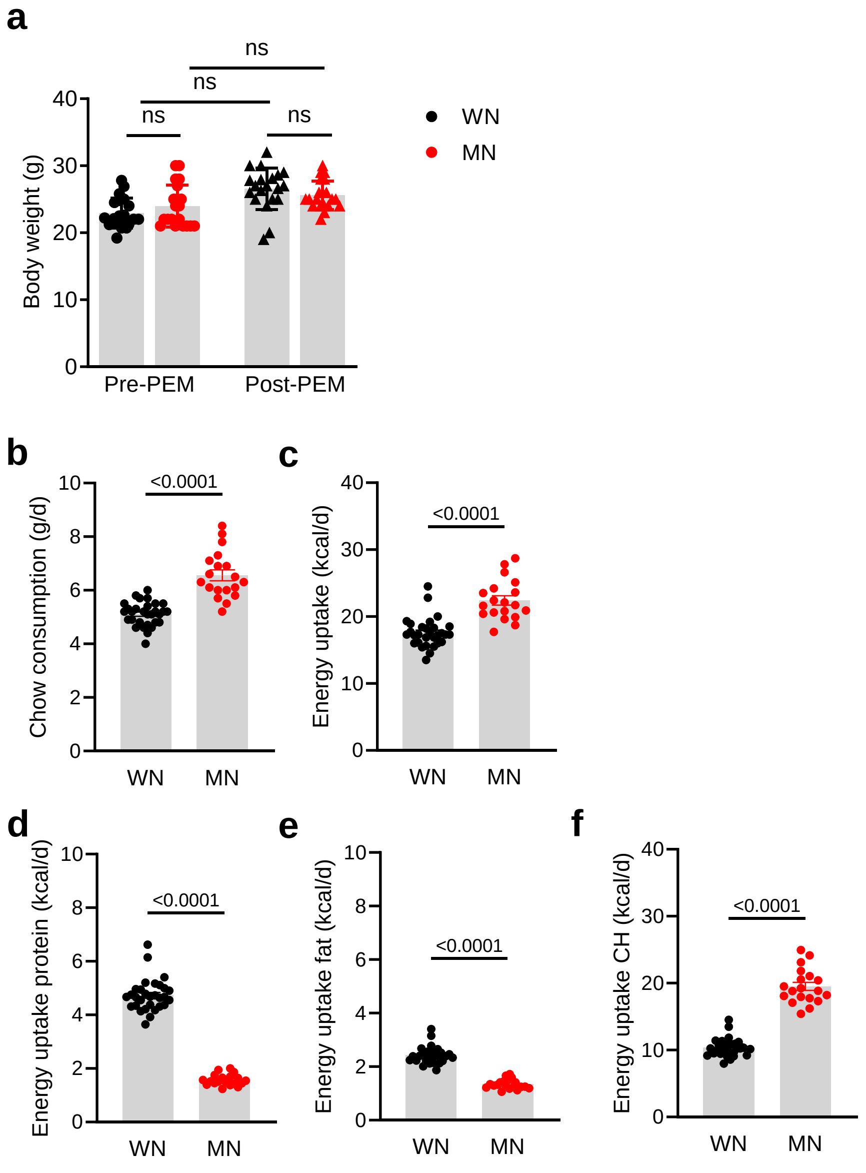

Supplementary Fig. 1 Body weight development and diet consumption and of WN and MN dams. a Body weights of WN and MN dams (*n*=20 each group) before start (Pre-MN) and at termination (Post-MN) of malnutrition in the MN group. Plots represent means ± SEM. *n.s.*, not significant (one-way ANOVA, *post hoc* Tukey's multiple comparison test). **b** Daily chow consumption in gram (g) and **c** daily energy uptake in kcal measured in WN dams (*n*=30) and MN dams (*n*=19). **d** Energy uptake by protein. **e** Energy uptake by fat. **f** Energy uptake by carbohydrates (CH). Plots represent means ± SEM. Exact p-values are displayed, ns, not significant (two-tailed MWU-tests).

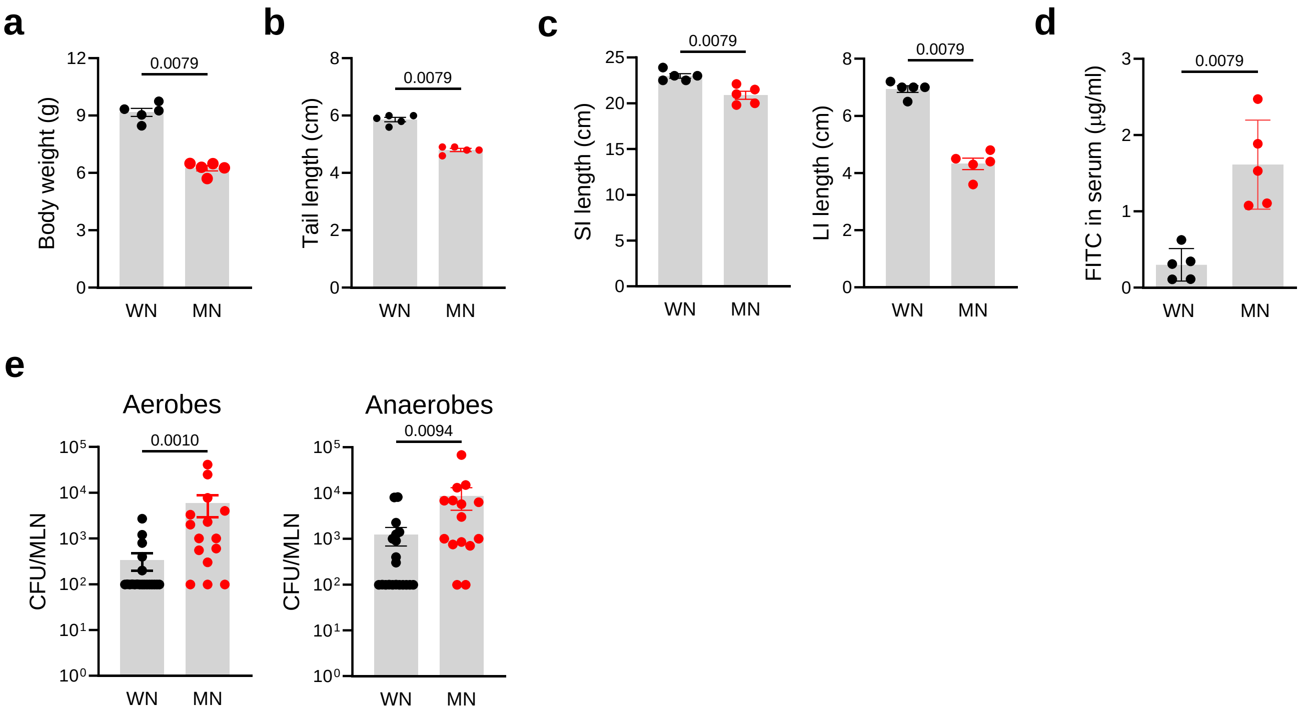

Supplementary Fig. 2 Maternal PEM causes stunting and gut mucosal barrier dysfunction in the offspring. The offsprings of WN and MN dams were assessed at d23 for signs of stunting (**a**-**c**) and gut mucosal barrier dysfunction (**d**,**e**). **a** Body weight. **b** Tail length. **c** SI and LI length (n=5 each group). **d** FITC-dextran levels in the serum (*n*=5 each group). **e** Number of aerobic or anaerobic bacteria in mesentery lymph nodes (MLN) plotted as colony-forming units (CFU) (WN: *n*=20, MN: *n*=15). Plots represent means ± SEM. Exact p-values are displayed (two-tailed MWU-tests).

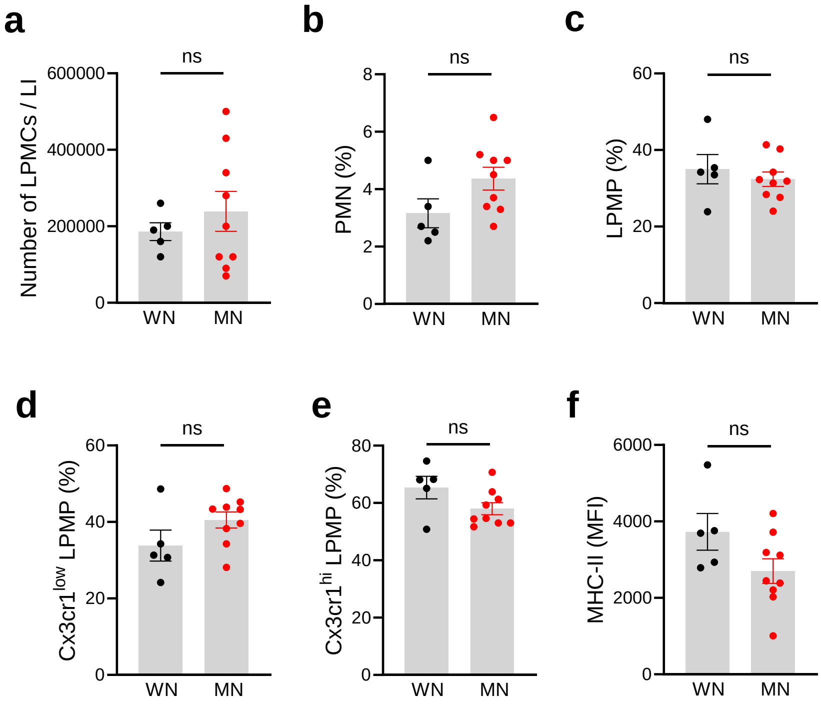

Supplementary Fig. 3 Maternal PEM during pregnancy does not induce intestinal inflammation in the fetus. a Number of LPMCs isolated from the LI collected from newborn WN and MN mice within 24h after birth. **b**-**f** Flow cytometric analysis of myeloid cells isolated from the LI of WN and MN neonates. **b**,**c** Proportions of PMNs (**b**) and LPMPs (**c**) from LPMCs. **d**,**e** Proportions of Cx3r1^{low} LPMPs (**d**) and Cx3r1^{hi} LPMPs (**e**) from LPMPs. **f** Expression of MHC-II on Cx3r1^{hi} LPMPs plotted as MFI. Plots represent means ± SEM. *ns*, not significant (two-tailed MWU-tests; WN: *n*=5, MN: *n*=9).

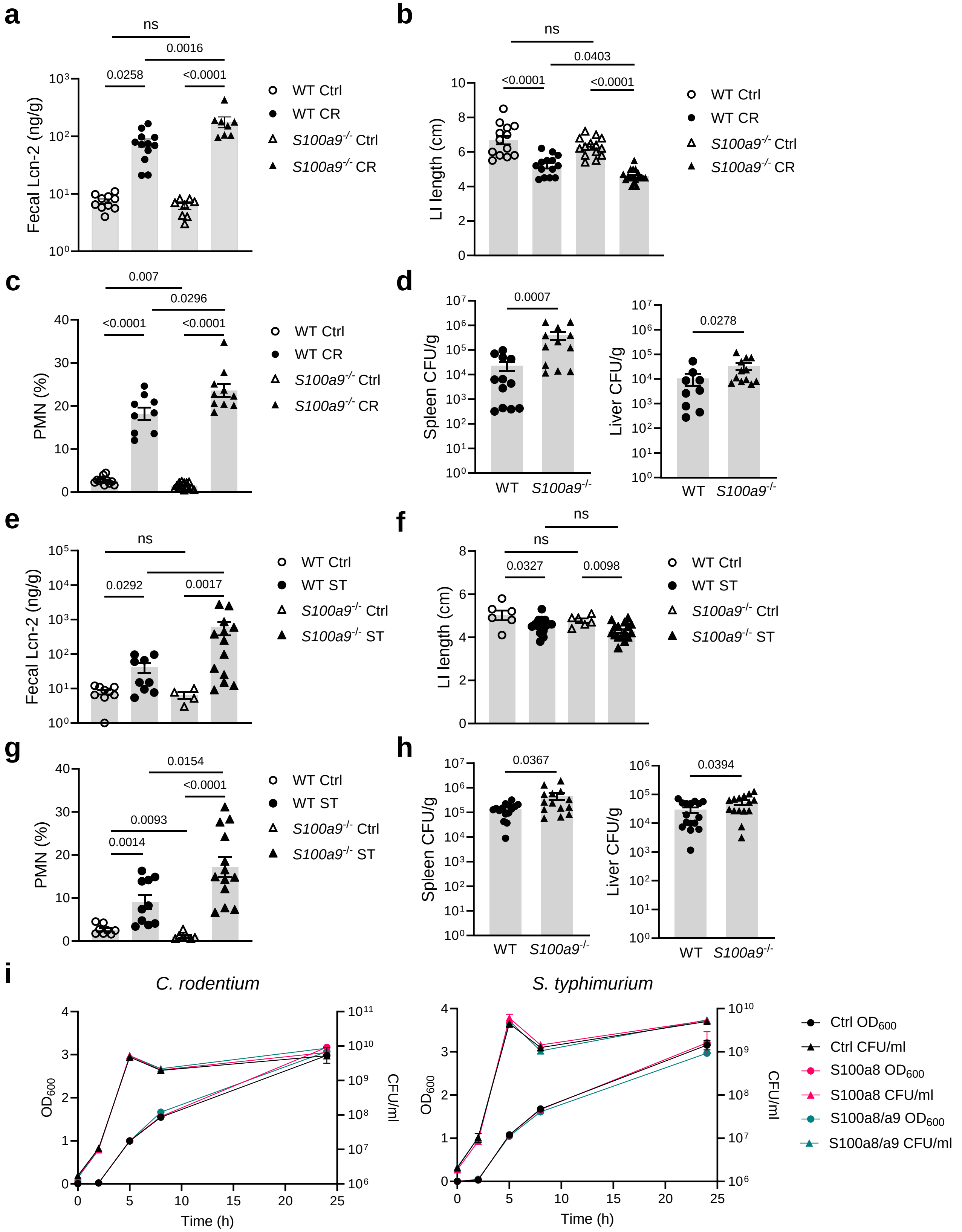

Supplementary Fig. 4 Increased susceptibility of *S100a9*$^{-/-}$ pups to enteric infections. a-d *S100a9*$^{-/-}$ mice and wildtype C57BL/6 mice (WT) were infected with *C. rodentium* (CR) or with PBS (control, Ctrl) at d12 after birth (WT Ctrl: *n*=10; WT CR: *n*=12; *S100a9*$^{-/-}$ Ctrl: *n*=8-10 *;* *S100a9*$^{-/-}$ CR: *n*=8-10). Biosamples were harvested 10 days p.i.. **e-h** *S100a9*$^{-/-}$ mice and WT mice were infected with *S. typhimurium* (ST) or treated with PBS (Ctrl) at d12 after birth (WT Ctrl: *n*=8; WT ST: *n*=10; *S100a9*$^{-/-}$ Ctrl: *n=6 ;* *S100a9*$^{-/-}$ ST: *n*=16). Biosamples were harvested 3 days p.i.. **a**,**e** Fecal Lcn2-levels. **b**,**f** LI length. **c**,**g** Proportions of PMNs from LI LPMCs. **d**,**h** Bacterial load in spleens and livers of infected mice plotted as CFU per organ weight. Plots represent means ± SEM. Exact p-values are displayed, *ns*, not significant (two-tailed MWU-tests. **i** *C. rodentium* and *S. typhimurium* were grown in the absence (Ctrl) and presence of S100a8/a9 (50 µg/ml) or S100a8 (5 µg/ml). OD_{600} values (left axis) and concentrations of colony forming units (CFU/ml, right axis) over culture times were plotted as mean ± S.D. (*n*=2 each group).

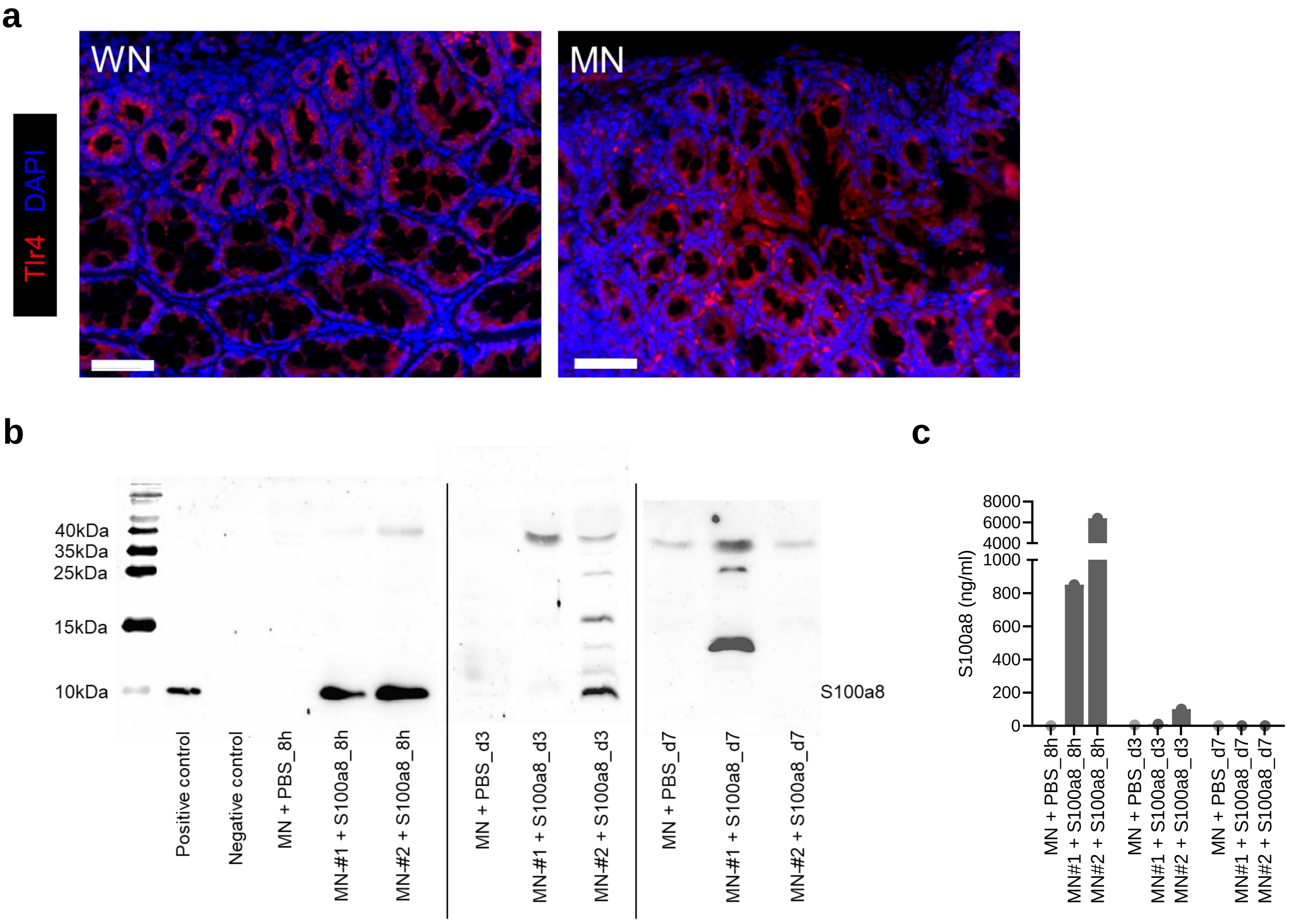

Supplementary Fig. 5 Tlr4 expression in the LI of WN and MN mice and bioavailability of S100a8 in MN mice after oral supplementation after birth. a Representative images of LI tissue samples from d7 WN mice (left) and d7 MN mice (right) immunostained for Tlr4 (red) and nuclei (DAPI; blue). Scale bars, 50 µm. **b,c** S100a8 immunoblotting of fecal sample extracts from MN mice fed PBS (MN + PBS, *n*=1)) and MN mice fed 5 µg of S100a8 (MN + S100a8, *n*=2) within 24h after birth, collected 8h, 3 days and 7 days after feeding, respectively. PBS with and without 5 µg of S100a8 served as positive respective negative control. S100a8 levels were semi-quantified in relation to the positive control (**c**).

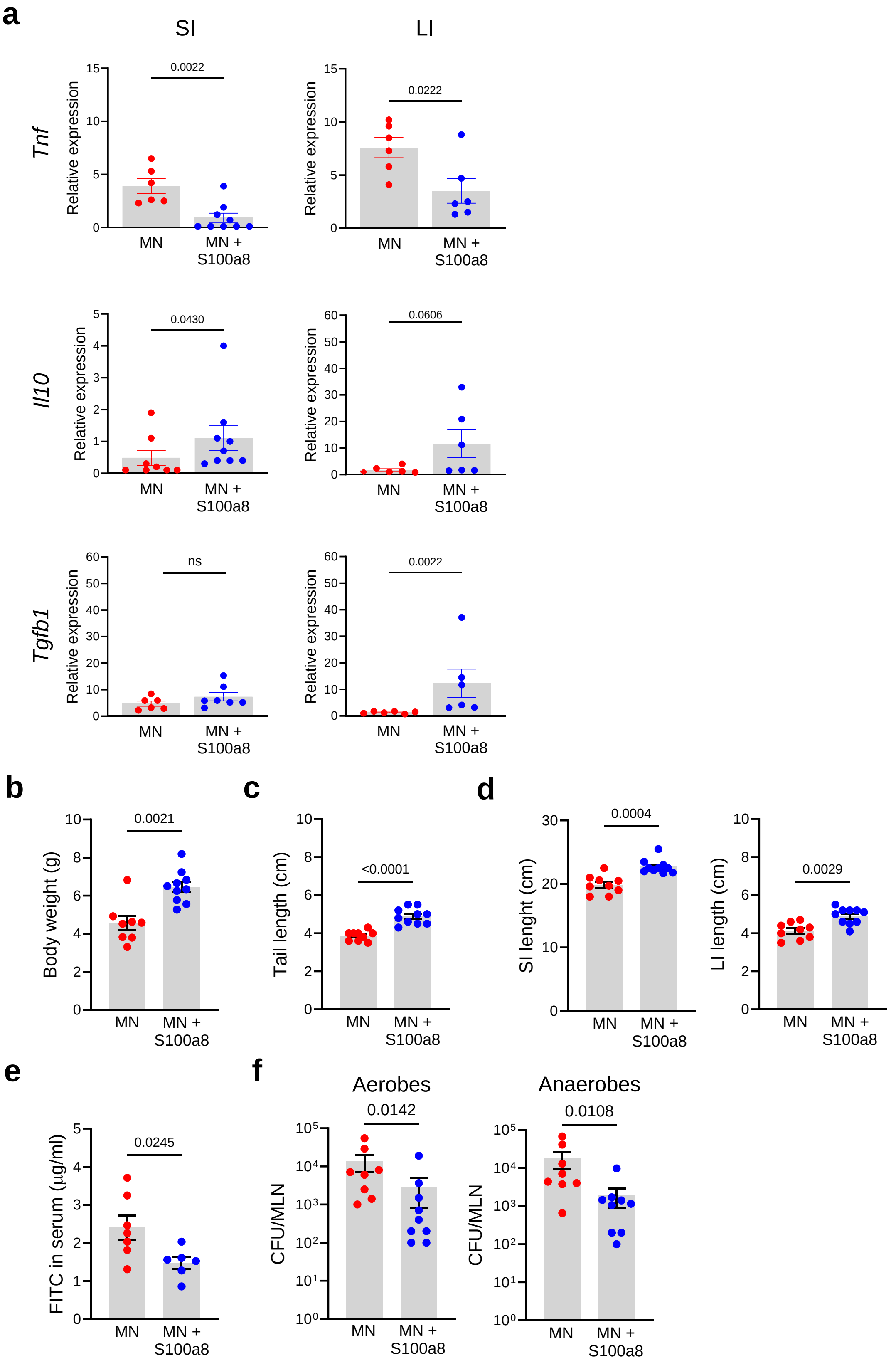

Supplementary Fig. 6 A singular feed of S100a8 after birth counteracts intestinal gene expression imbalances, overall growth failure and gut mucosal barrier dysfunction developing under maternal malnutrition. a-f Mice from MN dams were once supplemented after birth with S100a8 or left untreated. Mice were assessed at d23 for expression of indicated genes in LPMCs isolated from the SI and LI at d23 (*n*=6-9 each group) (**a**), signs of stunting (MN: *n*=9, MN+S100a8: *n*=10) (**b-d**) and barrier dysfunction (**e**,**f**). **b** Body weight. **c** Tail length. **d** SI and LI length. **e** FITC-dextran levels in the serum (MN: *n*=7 mice, MN+S100a8: *n*=6). **f** Number of aerobic or anaerobic bacteria in mesentery lymph nodes (MLN) plotted as colony-forming units (CFU) (MN: *n*=8 mice, MN+S100a8: *n*=9 mice) . Plots represent means ± SEM. Exact p-values are displayed, *ns*, not significant (two-tailed MWU-tests).

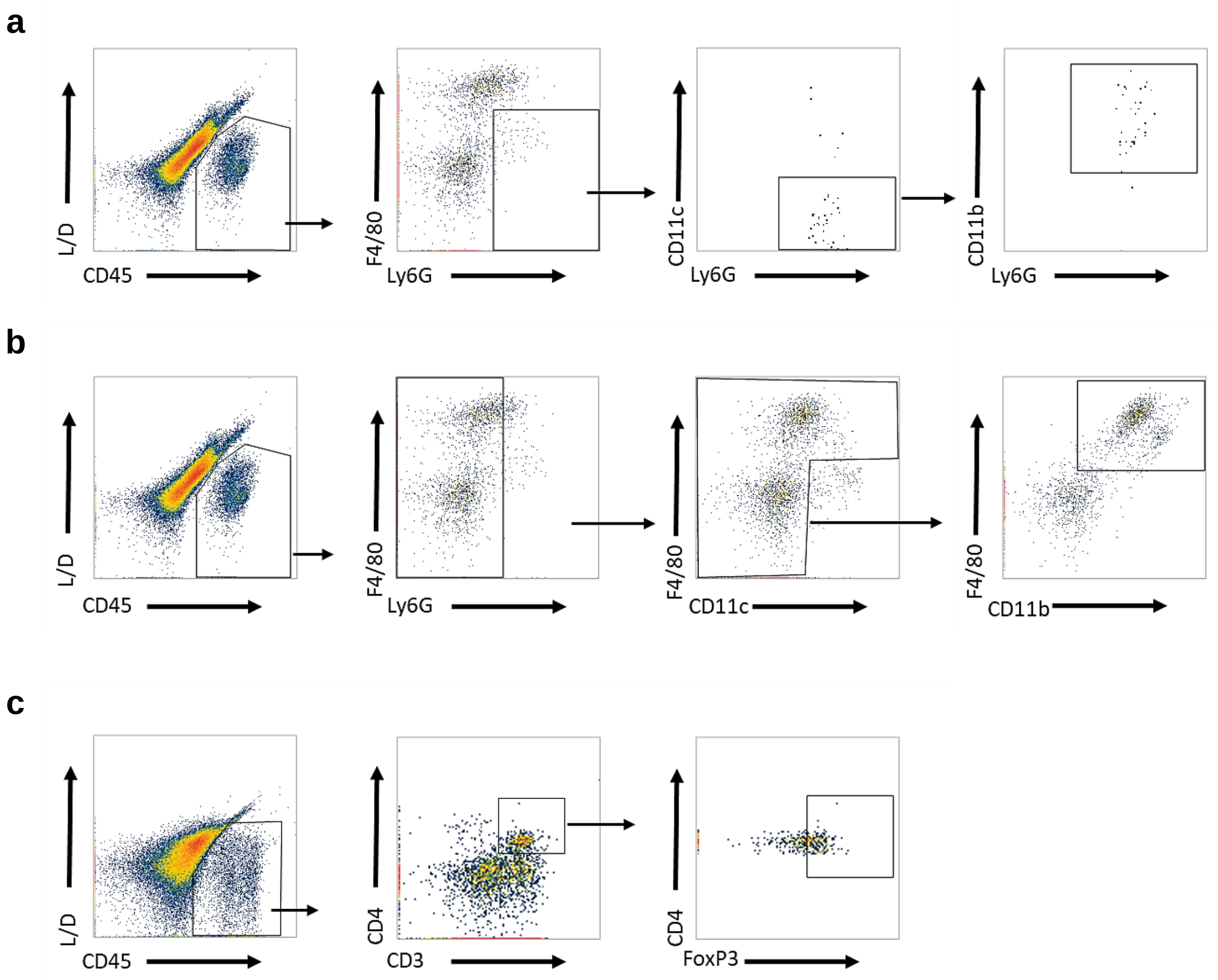

Supplementary Fig. 7 Gating strategies in the flow cytometric analysis of murine intestinal lamina propria cells. a-**c** After exclusion of cell debris, doublets and dead cells, CD45$^+$ leukocytes were gated according to the shown sequences in order to determine the proportions of CD11b$^+$Ly6G$^+$CD11c$^-$ PMNs (**a**), CD45$^+$F4/80$^+$CD11b$^{+/-}$Ly6G$^-$CD11c$^-$ LPMPs (consisting of yolk sac-derived F4/80^{hi} LPMPs and, with increasing age replacing, blood-derived F4/80^{low} LPMPs) (**b**), and CD3$^+$CD4$^+$FoxP3$^+$ Tregs (**c**) from LPMCs, respectively.
