# Supplementary material for: Postnatal supplementation with alarmins S100a8/a9 ameliorates malnutrition-induced neonate enteropathy in mice
Source: Nat Commun. 2024 Oct 4;15:8623. doi: 10.1038/s41467-024-52829-x (PMC11452687; doi:10.1038/s41467-024-52829-x)
Supplement: Supplementary file 3 — Description of Additional Supplementary Files [file 41467_2024_52829_MOESM3_ESM.docx]

Description of Additional Supplementary Files

File Name: Supplementary Data 1
Description: LDA effect size (LEfSe) in the WN versus MN group derived from 16S rRNA gene profiling of d23 caecum contents.

File Name: Source Data
Description: Raw data for every data point underlying all plots in the main and supplementary figures of this manuscript.
