# Supplementary material for: Postnatal supplementation with alarmins S100a8/a9 ameliorates malnutrition-induced neonate enteropathy in mice
Source: Nat Commun. 2024 Oct 4;15:8623. doi: 10.1038/s41467-024-52829-x (PMC11452687; doi:10.1038/s41467-024-52829-x)
Supplement: Supplementary file 5 — Reporting Summary [file 41467_2024_52829_MOESM5_ESM.pdf]

nature portfolio

Corresponding author(s): Dorothee Viemann, Lisa Peruzza

Last updated by author(s): Sep 10, 2024

Reporting Summary

Nature Portfolio wishes to improve the reproducibility of the work that we publish. This form provides structure for consistency and transparency in reporting. For further information on Nature Portfolio policies, see our Editorial Policies and the Editorial Policy Checklist.

Statistics

For all statistical analyses, confirm that the following items are present in the figure legend, table legend, main text, or Methods section.

n/a	Confirmed	
☐	☒	The exact sample size (*n*) for each experimental group/condition, given as a discrete number and unit of measurement
☐	☒	A statement on whether measurements were taken from distinct samples or whether the same sample was measured repeatedly
☐	☒	The statistical test(s) used AND whether they are one- or two-sided<br>*Only common tests should be described solely by name; describe more complex techniques in the Methods section.*
☐	☒	A description of all covariates tested
☐	☒	A description of any assumptions or corrections, such as tests of normality and adjustment for multiple comparisons
☐	☒	A full description of the statistical parameters including central tendency (e.g. means) or other basic estimates (e.g. regression coefficient) AND variation (e.g. standard deviation) or associated estimates of uncertainty (e.g. confidence intervals)
☐	☒	For null hypothesis testing, the test statistic (e.g. *F*, *t*, *r*) with confidence intervals, effect sizes, degrees of freedom and *P* value noted<br>*Give P values as exact values whenever suitable.*
☒	☐	For Bayesian analysis, information on the choice of priors and Markov chain Monte Carlo settings
☒	☐	For hierarchical and complex designs, identification of the appropriate level for tests and full reporting of outcomes
☒	☐	Estimates of effect sizes (e.g. Cohen's *d*, Pearson's *r*), indicating how they were calculated

Our web collection on statistics for biologists contains articles on many of the points above.

Software and code

Policy information about availability of computer code

Data collection	DIVA software v8.0.1 (BD Biosciences), QuantStudio Design & Analysis 2 Software (ThermoFisher), MiSeq reporter software v2.6 (Illumina)
Data analysis	FlowJo and FACS DIVA software v8.0.1 (both BD Biosciences), GraphPad Prism (v.10.1.0), iSeq reporter software v2.6 (Illumina), FastQC version 0.11.8 software , denoising algorithm of Callahan et al. (2016) , IQ-TREE stochastic algorithm Nguyen et al. (2015). Classification of sequences was performed using the Greengenes database gg_12_10.

For manuscripts utilizing custom algorithms or software that are central to the research but not yet described in published literature, software must be made available to editors and reviewers. We strongly encourage code deposition in a community repository (e.g. GitHub). See the Nature Portfolio guidelines for submitting code & software for further information.

Data

Policy information about availability of data

All manuscripts must include a data availability statement. This statement should provide the following information, where applicable:

- Accession codes, unique identifiers, or web links for publicly available datasets
- A description of any restrictions on data availability
- For clinical datasets or third party data, please ensure that the statement adheres to our policy

16S rRNA sequencing files were submitted to the NCBI Sequence Read Archive (www.ncbi.nlm.nih.gov/sra) and are available with BioProject accession number PRJEB68326 and PRJEB67750.

Research involving human participants, their data, or biological material

Policy information about studies with human participants or human data. See also policy information about sex, gender (identity/presentation), and sexual orientation and race, ethnicity and racism.

Reporting on sex and gender	N/A
Reporting on race, ethnicity, or other socially relevant groupings	N/A
Population characteristics	N/A
Recruitment	N/A
Ethics oversight	N/A

Note that full information on the approval of the study protocol must also be provided in the manuscript.

Field-specific reporting

Please select the one below that is the best fit for your research. If you are not sure, read the appropriate sections before making your selection.

- [x] Life sciences
- [ ] Behavioural & social sciences
- [ ] Ecological, evolutionary & environmental sciences

For a reference copy of the document with all sections, see nature.com/documents/nr-reporting-summary-flat.pdf

Life sciences study design

All studies must disclose on these points even when the disclosure is negative.

Sample size	For animal experiment, the a priori analysis demanding a power of 80% and a significance level of 5% while assuming a median effect of maternal malnutrition on the development of gut phenotypes and S100 breast milk levels over time (Cohen's d=0.25, single factor anova test) proposed sample sizes of n=26 per group to detect significant differences between groups (WN versus MN pups or S100a8-treated versus control-treated MN pups).
Data exclusions	No data needed to be excluded from the analyses.
Replication	All attempts at replicating the described experiments were successfull (data derived from at least 4 independent animal experiments from at least two litters) Moreover, the experiments were performed in two different laboratories, which in addition demonstrated also strong reproducibility.
Randomization	Both mouse strains (C57BL/6J, B6.S100a9tm1Nck) were constantly bred and litters were randomly used. Assingment to the expereimental groups of the dams was randomly done as well as the assingment of pups in the infection experiments.
Blinding	Investigators were not blinded to group allocation during data collection and analysis, as we aimed to investigate different experimental groups based age and nutritional state.

Reporting for specific materials, systems and methods

We require information from authors about some types of materials, experimental systems and methods used in many studies. Here, indicate whether each material, system or method listed is relevant to your study. If you are not sure if a list item applies to your research, read the appropriate section before selecting a response.

Materials & experimental systems

n/a	Involved in the study	
[ ]	[x]	Antibodies
[x]	[ ]	Eukaryotic cell lines
[x]	[ ]	Palaeontology and archaeology
[ ]	[x]	Animals and other organisms
[x]	[ ]	Clinical data
[x]	[ ]	Dual use research of concern
[x]	[ ]	Plants

Methods

n/a	Involved in the study	
[x]	[ ]	ChIP-seq
[ ]	[x]	Flow cytometry
[x]	[ ]	MRI-based neuroimaging

Antibodies

Antibodies used	Fixable Viability Dye eFluor 506 (1:1000, Cat. 65-0866-18, eBioscience), CD16/CD32 (1:100, 2.4G2, Cat. 101301, BioLegend), rat anti-mouse CD45 mAb (1:600, 30F-11, Cat. 11-0451-82, eBioscience), hamster anti-mouse CD11c mAb (1:400, N418, Cat. 45-0114-82, eBioscience), rat anti-mouse CD11b mAb (1:600, M1/70, Cat. A15390, eBioscience), rat anti-mouse Ly6G mAb (1:200, 1A8, Cat. 127617, BioLegend), rat anti-mouse F4/80 mAb (1:100, BM8, Cat. 17-4801-82 , eBioscience), mouse anti-mouse Cx3cr1 mAb (1:600, SA011F11, Cat. 149005, BioLegend), rat anti-mouse MHC-II mAb (1:600, M5/114.15.2, Cat. 743870, BD), hamster anti-mouse CD3e mAb (1:200, 145-2C11, Cat. 45-0031-82, eBioscience) rat anti-mouse CD4 mAb (1:600, RM 4-5, Cat. 47-0042-82 , eBioscience), rat anti-mouse FoxP3 mAb (1:100, FJK-16s, Cat. 17-5773-82, eBioscience), anti-murine S100a8 antibody (1:2500, purified by T.V.), anti-rabbit horseradish peroxidase seconday abntibody (1:5000, Cell Signaling), rat anti-mouse Ly6G (1:100, RB6-8C5, Santa Cruz), rabbit anti-mouse S100a9 polyclonal antibody (1:2500, purified by T.V.), rabbit anti-mouse Tlr4 polyclonal antibody (1:100, Novus), AlexaFluor488 goat anti-rat secondary anibody (1:1000, invotrogen), AlexaFluor555 donkey anti-rabbit secondary antibody (1:1000, invitrogen)
Validation	Clone numbers and/or catalog numbers are provided for all antibodies used for flow cytometry, immunofluoreszence and western blotting. Antibodies were previously validated by the manufacturer and citations are available on the manufacturer's website. All antibody stainings were titrated before usage in main experiments and compared to their respective isotype control.

Animals and other research organisms

Policy information about studies involving animals; ARRIVE guidelines recommended for reporting animal research, and Sex and Gender in Research

Laboratory animals	Mus musculus, strain C57BL/6 and B6.S100a9tm1Nck.<br>Mice were housed individually under controlled, specific pathogen free conditions (SPF) in barrier protected rooms with a 14 h light and 10 h dark cycle at two different animal laboratories, at the Hannover Medical School (Germany) and Institute for Research in Biomedicine (Switzerland). The room temperature was kept at 22±2°C with an air humidity of 55±5%.
Wild animals	N/A
Reporting on sex	Both sexes were used equally and subsetted where appropriate.
Field-collected samples	This study does not involve field-collected samples.
Ethics oversight	Mouse experiments were performed according to the Swiss Federal Veterinary Office guidelines and the German Animal Welfare Legislation and has been approved by the Cantonal Veterinary Office (approval no. 31975 TI-19/2020/2023; 34029 TI-40/2021; 34591 TI-02/2022) and the Lower Saxony State Office for Consumer and Food Safety, Germany (approval no. 20/3366; 21/3683).

Note that full information on the approval of the study protocol must also be provided in the manuscript.

Plants

Seed stocks	No plants.
Novel plant genotypes	No plants.
Authentication	No plants.

Flow Cytometry

Plots

Confirm that:

- [x] The axis labels state the marker and fluorochrome used (e.g. CD4-FITC).
- [x] The axis scales are clearly visible. Include numbers along axes only for bottom left plot of group (a 'group' is an analysis of identical markers).
- [ ] All plots are contour plots with outliers or pseudocolor plots.
- [x] A numerical value for number of cells or percentage (with statistics) is provided.

Methodology

Sample preparation	FACS analysis was performed using single-cell suspensions of SI and LI LPMC. In all staining panels, the Fixable Viability Dye eFluor 506 (eBioscience) was used for the exclusion of dead cells. CD16/CD32 (2.4G2, BioLegend) was used for blocking purposes. All stainings were performed for 30 minutes in the dark at 4°C. After surface staining, cells were fixed using 2% PFA. Subsequently, cells were stained in Intracellular Staining buffer (ISB, FACS buffer with 0.5% saponin and 0.2% Tween20) for another 30 min at 4°C in the dark.
Instrument	All flow cytometry analyses were performed using a FACS Canto II (BD Biosciences) flow cytometer.
Software	Data were analyzed using the FlowJo software or FACS DIVA software v8.0.1 (both BD Biosciences).
Cell population abundance	N/A, sorting has not been performed.
Gating strategy	Gateing strategy is illustrated in Supplementary Fig. 5.<br>PMN: After exclusion of cell debris (foward/side scatter), doublets (foward scatter / height plots) and dead cells (dye), CD45+ leukocytes were further gated as follows: CD45+CD11b+Ly6G+CD11c- cells.<br>LPMP: After exclusion of cell debris (foward/side scatter), doublets (foward scatter / height plots) and dead cells (dye), CD45+ leukocytes were further gated as follows: CD45+F4/80+CD11b+/-Ly6G-CD11c- cells.<br>Tregs: After exclusion of cell debris (foward/side scatter), doublets (foward scatter / height plots) and dead cells (dye), CD45+ leukocytes were further gated as follows: CD3+CD4+FoxP3+ cells.

☒ Tick this box to confirm that a figure exemplifying the gating strategy is provided in the Supplementary Information.
